# Unearthing Genetic Treasures: Exploring Lost Autochthonous *Vitis vinifera* Varieties in Lebanon

**DOI:** 10.3390/genes15121617

**Published:** 2024-12-17

**Authors:** Carole Saliba, Alba María Vargas, María Teresa de Andrés, Françoise Lamy, Liliane Boukhdoud, Rhea Kahale, Thierry Robert, Rani Azzi, Noel Abinader, Magda Bou Dagher Kharrat

**Affiliations:** 1 Laboratoire Biodiversité et Génomique Fonctionnelle, Faculté des Sciences, Université Saint-Joseph, Campus Sciences et Technologies, Mar Roukos, Mkalles, P.O. Box 1514, Riad el Solh, Beirut 1107 2050, Lebanon; carole.saliba@net.usj.edu.lb (C.S.); liliane.boukhdoud@net.usj.edu.lb (L.B.); rhea.kahale@net.usj.edu.lb (R.K.); 2 Instituto Madrileño de Investigación y Desarrollo Rural, Agrario y Alimentario (IMIDRA), Finca El Encín, Ctra. A2, Km 38200, 28805 Alcalá de Henares, Spain; alba.vargas@madrid.org (A.M.V.); maite.deandres@madrid.org (M.T.d.A.); 3 Laboratoire ESE—UMR 8079, Université Paris-Saclay, CNRS, AgroParis Tech, IDEEV, 91190 Gif-sur-Yvette, France; francoise.lamy@universite-paris-saclay.fr; 4 Laboratoire Ecologie Systématique et Evolution, IDEEV, Université Paris-Saclay, 91190 Gif-sur-Yvette, France; thierry.robert@universite-paris-saclay.fr; 5 Vineyard and Wine Industry, 34 Rue Schnapper, 78100 Saint-Germain-en-Laye, France; azzi.rani@gmail.com; 6 Venturis S.A.L., Elmir Brewery Building, Charles Helou Highway, 900073 Jdeideh, Lebanon; contact@venturis.com

**Keywords:** *Vitis vinifera*, Lebanon, grapevine, SSR markers, autochthonous, genetic diversity

## Abstract

Background/Objectives: Lebanon, one of the oldest centers of grapevine (*Vitis vinifera* L.) cultivation, is home to a rich diversity of local grape varieties. This biodiversity is linked to the country’s unique topography and millennia of cultural history. However, the wine industry primarily utilizes international varieties, putting many local varieties at risk of extinction. Methods: In this study, we analyzed 202 samples from old vineyards, home gardens, and private collections using 21 microsatellite markers to assess their identity and genetic diversity. Results: A total of 67 different genotypes were identified, with 34 not matching any existing profiles in the consulted databases, based on comparisons with the European Vitis Database, the Vitis International Variety Catalogue (VIVC), and the databases established in two previous studies conducted in Armenia and Lebanon. Cluster analyses revealed Lebanon’s rich diversity of local grape varieties, highlighting cases of synonymy, homonymy, and misnaming. All loci were polymorphic, with 228 alleles and an average of 11.4 alleles being detected. The highest number of alleles was observed at the VVIV67 locus (19 alleles), while the lowest was found at the VVIQ52 and VVIN73 loci (5 alleles). The observed heterozygosity was 0.732, slightly below the expected value of 0.757, with gene diversity varying among the markers. Conclusions: Of the 67 genetic profiles identified, 34 are absent from national and international databases, underscoring Lebanon as a hotspot for grapevine genetic diversity. This unique genetic variation, which includes several synonyms due to geographic isolation, could provide valuable opportunities for producing distinctive wines and emphasizes the need for further research and documentation.

## 1. Introduction

*Vitis vinifera* ssp. *vinifera* includes numerous grape varieties shaped by centuries of cultivation, significantly influencing its phenotype and biology, which are crucial for winemaking. Wine production in the Eastern Mediterranean dates back 7000 to 8000 years [1]. Grape vines migrated westward to North Africa and Western Europe and eastward toward China and Japan along the Silk Road [2]. They appeared in Greece and Egypt around 4500 years ago and reached Italy approximately 3000 years ago. Grape cultivation in southern France likely began in the Greek city of Massalia about 2600 years ago [3], coinciding with Phoenician arrival in the Iberian Peninsula [4]. The spread of grapevines is linked to Greek and Phoenician expansion [5,6]. Phoenician influence significantly impacted viticulture in Spain, although local populations had used grapevines since the Neolithic period [7]. As grapevines spread to new regions, they frequently hybridized with local wild populations, enriching their genetic diversity [8]. The vast number of grape varieties resulted from both natural processes—such as local adaptation and hybridization—and human interventions like selective breeding and historical trade routes. Currently, among the 10,000 known grapevine varieties, 13 account for over one-third of the global vineyard area, while 33 varieties represent 50% [9].

Lebanon has long been a vital center for viticulture, with its ancient terroirs and winemaking traditions dating back millennia [10,11]. Renowned for producing the “nectar of the gods” and “golden wine”, Lebanese winemaking is deeply rooted in the country’s cultural heritage. The ancient Phoenicians, based along Lebanon’s coast, developed maritime trade routes, sharing their viticultural knowledge throughout the Mediterranean. Archeological evidence, such as ancient pottery (amphorae) found in Byblos and Tell el-Burak, highlights early wine production [12]. Additional discoveries, including ancient wine presses and terraced vineyards, provide insight into historical winemaking practices. The Temple of Eshmun in Sidon also contains artifacts related to wine offerings [13]. Lebanon’s diverse climate, isolated valleys, and varied soil types have fostered rich grapevine biodiversity, making it a hotspot for grape varieties. Although the exact number of local varieties is uncertain, this diversity significantly contributes to Lebanon’s unique winemaking traditions and its standing in the global wine industry.

In 1814, Charles Lewis Meryon, an English physician, documented 21 Lebanese grape varieties and vinification methods in the Machmouché area. Notable local varieties include ‘Obeidi’, ‘Merwah’, and ‘Mekssessé’, as well as ancient red grapes like ‘Assouad Karech’ and ‘Asmi Noir’ [14,15]. Commercial table grape plantations primarily featured ‘Obeidi’, ‘Beitamouni’, ‘Tfayfihi’, and ‘Maghdouche’.

The modern wine industry began in 1857 with French Jesuits establishing vineyards in Ksara, and the first commercial wine was produced by François Eugène Brun a decade later. The French mandate from 1920 revived viticulture, introducing Western grape varieties, such as ‘Syrah’ and ‘Cabernet Sauvignon’, which led to a decline in local varieties [14]. The introduction of Western cultivars and the phylloxera outbreak in 1910 resulted in a homogenization of grape varieties and a loss of local identity [16,17]. Replanting with resistant rootstocks may have altered the character of Lebanese wines, impacting their identity and authenticity [18,19,20,21]. Although commercial vineyards thrive with international varieties, family-run vineyards in Mount Lebanon maintain traditional grape varieties, preserving a glimpse of Lebanese winemaking heritage. There is a growing global movement to recognize and value local grape varieties for their heritage and economic potential [22]. Protecting and recovering these varieties is crucial for adapting to climate change and maintaining economic viability. Lebanese growers are increasingly replanting indigenous varieties, reconnecting with their historical roots and enhancing the uniqueness of their wines, while also addressing climate resilience and leveraging terroir for marketing. In addition to their cultural significance, Lebanese grape varieties may also play a vital role in adapting to climate change. The Mediterranean region is already experiencing severe impacts, including a 30% decrease in precipitation and increased heat waves [23,24], with forecasts predicting further changes in rainfall and temperature, as well as more frequent droughts [25]. These climatic challenges could reduce both yield and wine quality [26]. In this context, the diverse cultivars of *V. vinifera* are crucial; exploring their inter- and intra-varietal diversity may enhance adaptability to climate shifts, emphasizing the need for resistance and sustainability [27,28]. Research indicates that local grape varieties often outperform more common ones in resisting abiotic stress and diseases [29,30]. However, establishing the origins of local grape varieties in Lebanon is challenging due to a lack of genetic studies and archeological documentation. Characterizing and identifying neglected native varieties is essential for preserving Lebanon’s winemaking identity and understanding genetic diversity and relationships among genotypes. Recovering these autochthonous varieties can help prevent diseases and enhance adaptability to soil and climate conditions [31], while also allowing for better responses to market demands [25]. Despite their importance, identification and conservation efforts are complicated by the limited genetic research and the subjective nature of traditional ampelographic methods, which can be influenced by environmental factors [32]. Ampelography has certain limitations, including the potential for misidentification and the inability to capture the full extent of genetic diversity within grape varieties. Consequently, it is often supplemented with molecular techniques and genetic analyses to provide a more holistic understanding of grapevine diversity and the relationships between different varieties [33]. Molecular characterization using Simple Sequence Repeat (SSR) markers offers a reliable method for identifying grapevine cultivars, assessing genetic relationships, and conducting parentage analysis [34,35,36]. SSR markers have been widely used to identify and genotype grapevine cultivars collected from old vineyards in the Mediterranean region, such as those in Montenegro [37]. They have also been used to determine a region’s autochthonous grapevine cultivars [38]. Moreover, SSRs have proven essential in studying the genetic diversity and genetic relationships between grape varieties [39,40,41]. For example, a study conducted in Cyprus used 11 SSR markers through molecular genotyping, enabling the precise identification and discrimination of native grape varieties. This analysis also shed light on their genetic relationships with *Vitis* material from Greece, Bulgaria, and Western Europe [42]. Likewise, the utilization of DNA typing with 13 SSR markers revealed the presence of 28 distinct genotypes, primarily consisting of native grapevine varieties cultivated within the archipelago of Malta; this significantly contributed to the precise identification of previously unidentified or neglected grapevine genetic resources in the region [43]. SRRs were also used to achieve a precise identification of Sardinian indigenous grapevine cultivars and to effectively address challenges related to synonymous names, homonyms, and incorrect naming [44]. At the national level, Lebanon is home to over 27 native grape varieties, primarily white table grapes, according to the VIVC. In 1998, the Lebanese Agricultural Research Institute (LARI) established the first grapevine genetic resources repository, marking a significant step in conserving Lebanon’s native grapevine varieties [45]. While subsequent surveys of grapevine-producing areas have primarily relied on morphological assessments [15,46], molecular characterization using ISSR markers has revealed considerable genetic diversity [45]. Despite these findings, limited knowledge exists regarding the full extent of Lebanon’s local grape germplasm, emphasizing the need for further molecular research to better understand and conserve this genetic diversity.

The objective of this study is to characterize and identify grapevine cultivars in Lebanon, focusing on local varieties. Genetic relationships among genotypes will be assessed using SSR marker profiles to discover and preserve native cultivars. This research is vital for maintaining the unique identity of Lebanese wines and addressing shifts in market demands. Additionally, the findings will help identify new synonyms, homonyms, and errors, showcasing the distinctiveness of varieties cultivated in Lebanon. To achieve these objectives, 202 grapevine samples from old vineyards, home gardens, and private collections in Lebanon were analyzed using 21 microsatellite markers to assess genetic diversity and identity. All loci were polymorphic, detecting 228 alleles, with an average of 11.4 alleles per locus. A total of 67 different genotypes were identified, 34 of which did not match any existing profiles in international databases. As one of the first comprehensive genetic analyses of Lebanese grape germplasm, this study underscores the need for further research and collaboration with national germplasm repositories to enhance the identification and categorization of unique varieties. In summary, our study has uncovered a previously undocumented genetic landscape of Lebanese grapevine varieties, emphasizing the importance of this diversity for grapevine preservation and viticultural research.

## 2. Materials and Methods

### 2.1. Sample Collection

**Figure 1:**
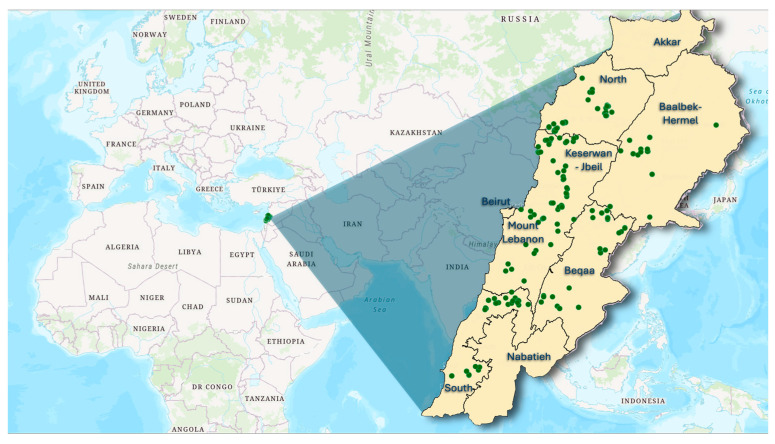
Geographic distribution of sampling sites in Lebanon represented by green dots.

From 2020 to 2021, a total of 202 grape leaf samples were collected for molecular analysis from 101 locations across six governorates in Lebanon. The distribution of samples varied by region (Figure 1), with North Lebanon contributing the largest sample size of 52 accessions, followed by Mount Lebanon with 36 accessions. The Beqaa region yielded 33 accessions, Kesrouane Al Fatouh–Jbeil accounted for 32 accessions, South Lebanon provided 30 accessions, and the Baalbek–Hermel region had the lowest sample size with 19 accessions. Samples were obtained from both old and abandoned vineyards as well as domestic gardens, and specific variety names were recorded whenever possible. Each sampling site was documented with GPS coordinates and elevation data, ranging from 50 m to 1600 m above sea level, reflecting the diverse agricultural climatic conditions throughout Lebanon. A comprehensive list of the collected accessions, including their codes and vernacular names, is provided in Supplementary Table S1. Additionally, the study included six standard cultivars, ‘Chardonnay’, ‘Aswad Karech’, ‘Merlot’, ‘Syrah’, ‘Cabernet Sauvignon’, and ‘Obeidi’, which served as reference points for molecular comparisons.

### 2.2. DNA Extraction and nSSR Analysis

After collection, the leaves were dried and preserved in silica gel until extraction. Subsequently, the leaves were ground into a fine powder using a mortar and pestle with the aid of liquid nitrogen. DNA extraction from the plant powder was carried out utilizing the DNeasy Plant Mini Kit from Qiagen (Hilden, Germany), following the recommended procedures provided by the manufacturer. To assess the quality of the extracted DNA, electrophoresis was performed using 1% agarose gel.

For genotyping, microsatellite fingerprinting was conducted on 21 microsatellite loci (nSSRs). These loci were selected based on the study by Margaryan et al. in 2021 [47] (Supplementary Table S2) and were strategically distributed across the sixteen grape chromosomes as previously described. The specific microsatellite loci used were as follows: VVS2, VVMD5, VVMD7, VVMD24, VVMD25, VVMD27, VVMD28, VVMD32, four from the VrZAG series (VrZAG62, VrZAG79, VrZAG67, and VrZAG83), VMC1b11, and eight from the VVI series (VVIb01, VVIn16, VVIh54, VVIn73, VVIp60, VVIv37, VVIv67, and VVIq52).

To identify matching profiles and verify synonyms and homonyms, fingerprints from the European Vitis Database, accessible at www.eu-vitis.de, were used. Additional accessions were also incorporated from the Vitis International Variety Catalogue (VIVC) database, accessible at https://www.vivc.de/ (accessed on 1 September 2023), as well as accessions from Laboratorio de Genotipado de Vid, Spain, and the Armenian database (communicated personally). Nine polymorphic microsatellite markers recommended by the GrapeGen06 project [48], specifically VVMD5, VVMD7, VVMD25, VVMD27, VVMD28, VVMD32, VVS2, VrZAG62, and VrZAG79, were employed to compare the genetic profiles with the SSR-marker database, whereas the whole set of 21 SSR markers was utilized for comparisons with the Armenian database and Spanish accessions.

Fragment length determination using capillary electrophoresis was carried out in the GENTYANE (GEnoTYping and sequencing in AuvergNE) platform in Clermont-Ferrand. In this process, all forward primers were labeled with fluorescent dyes (specifically FAM, HEX, TAMRA, ROX, and ATTO 565) at their 5′ ends. By using markers with different labels and varying fragment lengths, polymerase chain reaction (PCR) was conducted, and markers were grouped into six multiplex pools. Each pool consisted of two to five SSR markers with similar annealing temperatures (Supplementary Table S2). The QIAGEN^®^ Multiplex PCR Kit (Qiagen, Hilden, Germany) was employed to set up 25 µL reaction mixtures, comprising 2X Qiagen Multiplex PCR master mix (Qiagen, Hilden, Germany), 10X primer mix (Eurofins Genomics, Ebersberg, Germany) (2 μM each primer), and 1 ng of template DNA. The amplification process was conducted using the Eppendorf Mastercycler^®^ nexus GSX1 (Eppendorf, Hamburg, Germany) PCR Thermal Cycler with the following program: an initial denaturation step of 5 min at 95 °C, followed by 30 amplification cycles of 30 s at 95 °C, 1 min and 30 s at 58 °C, 30 s at 72 °C, and a final extension step of 7 min at 72 °C. After the PCR, the results of fragment length determination were analyzed using the GeneMapper 4.0 software from Applied Biosystems, Life Technologies, Waltham, MA, USA. The fragment sizes were recorded in base pairs. To determine allele sizes, the fragment peaks were compared with the internal size standard LIZ500, employing the Microsatellite default method for size calling with SSRs and the expected repeat size. To account for any amplification shifts that might have occurred among different multiplexes, six standard cultivars were used in each PCR amplification run. These same cultivars were used to convert the alleles to be able to compare with other databases in order to identify the varieties.

### 2.3. Genetic Diversity Analysis

The genetic diversity statistics, including allele size range (Ra), the number of different alleles (Na), the number of effective alleles (Ne), Shannon’s Information Index (I), observed heterozygosity (Ho), expected heterozygosity (He), and the fixation index (Fis), also known as the inbreeding coefficient, were computed using GenAlEx software version 6.5 [49,50] to evaluate the genetic diversity within the Lebanese grape germplasm. Furthermore, allele frequencies for each SSR marker were calculated. Clustering was performed using MEGA 11 software [51], version 11.0.13. A distance tree was generated using the neighbor-joining (N-J) hierarchical clustering method based on the pairwise Euclidean distance derived from the genetic distance obtained in GenAlEx software.

## 3. Results

### 3.1. Identification of Samples

In this study, 202 grape leaf samples were collected for molecular analysis from 101 locations across six governorates in Lebanon. Notably, the distribution of accessions varied by location, with Abdelli (ABD) exhibiting the highest representation at 12 samples, followed closely by Ablah (ABH) with 9 individuals, Boul (BOU) with 7 individuals, and Blawza (BLA) with 6 individuals.

The accessions analyzed were associated with 24 different vernacular names, as initially reported by the collectors. ‘Hifawi’ was the most common, with seven samples, followed by ‘Tfaifihi’ and ‘Beitamouni’, each with six samples, ‘Merwah’ with five, and ‘Meksassi’ with four. This diversity in naming highlights the complex grape naming conventions in Lebanon, which are often based on factors such as variety (characterized by flavor, color, and shape), region of cultivation, or traditional nomenclature. It is particularly noteworthy that a substantial number of samples (151) were not assigned specific names and were categorized as lacking data or designated by the color of the grape bunch (red or white). This underscores the challenges in documenting the genetic diversity of these populations and the potential loss of traditional knowledge associated with grape cultivation. It highlights the importance of engaging with local communities to gather more information about these varieties, as they may represent valuable genetic resources that have yet to be documented. To further explore this diversity, the analysis involved genotyping the collected samples using SSR markers.

The analysis involved genotyping 202 samples using 21 SSR markers; however, the marker VVIV37 was excluded due to the absence of detectable profiles across all samples. Ultimately, profiles were successfully obtained for 202 accessions, with detailed genetic profiles available in Supplementary Table S3. Among the 202 accessions, redundant samples were efficiently identified using the remaining 20 SSR markers. Accessions exhibiting the same SSR profile were classified as redundant genotypes, with 135 individuals forming these redundant groups. This finding highlights the prevalence of certain genetic lineages within the population. Notably, identical profiles—indicative of the same cultivars—were observed even in geographically distant locations.

This study identified a total of 67 genotypes, encompassing Lebanese varieties, internationally recognized varieties, and regional varieties from neighboring areas such as Syria, Turkey, and Israel. Additionally, some accessions did not match any profiles in the European Vitis Database, the VIVC database, the Laboratorio de Genotipado de Vid in Spain, or the Armenian database, and were thus classified as “unmatched”. Supplementary Table S4a highlights the matches between the genetic profiles of our samples and those in the databases, including the VIVC variety number, name, and origin, while Supplementary Table S4b lists the redundant genotypes identified in this study.

After comparing the SSR profiles of our samples with those in the aforementioned databases, we successfully assigned 33 genotypes corresponding to 143 collected samples to known cultivars. This genetic profiling provides a comprehensive understanding of the existing genetic variants within the population. Among these, ten genotypes matched internationally recognized varieties and rootstocks, including ‘Millardet et Grasset 41-B’, ‘Syrah’, ‘Muscat Hamburg’, ‘Kober 5BB’, ‘Chardonnay’, ‘Millardet et Grasset 420 A’, ‘Crimson Seedless’, ‘Red Globe’, ‘Lattuario Nero’, and ‘Ganzin 1′. Additionally, eleven genotypes aligned with regional varieties, such as ‘Assoued Kere’, ‘Dabouki’, ‘Maa’tar’, ‘Blanc De Dellys’, ‘Horoz Karasi’, ‘Gallurazeni Di Damasco’, ‘Asswad Zeitouni’, ‘Asswad Abou Khisle’, and ‘Verico’. Five genotypes were classified as distinct Lebanese varieties: ‘Beitamouni’, ‘Asswad Karech’, ‘Obeidi’, and ‘Zeitouni’. Additionally, our results were compared with the study conducted by Merheb et al. [52] on underexploited Lebanese grapevine resources, where seven genotypes were found to be common between both studies. Altogether, 12 Lebanese genotypes were identified. Importantly, ‘Blanc De Dellys’ was the most frequent variety in our analysis, identified in 29 samples, followed by ‘Dabouki’, which appeared in 27 samples. These findings illustrate the complex genetic landscape of the grapevine population and underscore the importance of conservation efforts for these diverse genetic resources.

However, among the 67 identified genotypes, 34 unique genotypes corresponding to 59 samples remained unidentified as they did not match any entries in the databases. This underscores the novel genetic diversity present within the studied population. This poses significant challenges for genetic analysis, particularly for samples with fewer than 40 alleles, which are represented by only one sample. Moreover, the absence of adequate reference data for these unidentified samples hinders reliable comparisons with known genotypes, complicating the determination of their true identities.

Notably, within the 34 unidentified genotypes, 26 unique genotypes were detected, each represented by a single sample. Furthermore, 41 samples corresponded to 25 genotypes, all of which exhibited complete genetic profiles characterized by 40 alleles that did not match any existing profiles in the consulted databases. This significant finding underscores the potential of these samples to represent “previously uncharacterized indigenous genotypes”. The absence of matches suggests that these genotypes may be unique and previously undocumented, emphasizing their importance as novel, indigenous grape varieties. Given their genetic distinctiveness, these samples warrant further investigation to explore their unique traits and contributions to grapevine diversity. Conservation efforts are essential to preserve these genotypes, as they may hold valuable characteristics for future viticulture, including resilience to pests, adaptability to different climates, and unique flavor profiles. Interestingly, our analysis of the unidentified samples revealed two genotypes with consistent genetic profiles across multiple samples, suggesting they may represent ancient, previously uncharacterized varieties. The first genotype (G6) displays identical genetic profiles in seven samples, each containing 40 alleles, suggesting a significant historical lineage. Similarly, the second genotype (G56) exhibits genetic uniformity in five samples, also with 40 alleles, indicating its potential as an ancient variety with unique genetic traits. These findings underscore the complexity and richness of genetic diversity within the grape population, providing valuable insights into evolutionary processes and highlighting the importance of conservation strategies for preserving viticultural heritage.

While genetic diversity is a key factor, the geographical origin of grapevine samples also influences the redundancy and distribution patterns of genotypes in Lebanon. Frequent genotypes, such as ‘Blanc de Dellys’ and ‘Dabouki’, were identified across multiple Lebanese locations, highlighting their adaptability to diverse environments and their historical significance in local viticulture. The widespread presence of these genotypes in geographically distant areas likely results from human-mediated propagation, facilitated by historical trade routes and agricultural practices. In contrast, single unmatched genotypes were predominantly found in isolated, high-altitude regions such as Mrouj and Ehden. The environmental isolation of these areas may have acted as a natural barrier, preserving distinct genetic profiles and protecting these varieties from crossbreeding with more common genotypes. Regarding redundancy, some identical genotypes were clustered within the same region (e.g., G3, G15 and G70), while others, like G7 and G28, were found in nearby sites, suggesting localized propagation practices within specific areas. Interestingly, some redundant genotypes (e.g., G56 and G6) were found across multiple, geographically distant regions, further supporting the idea that human-mediated dissemination or historical trade may explain their widespread presence. These findings underscore the intricate interplay between local adaptation, historical dissemination, and geographical isolation in shaping Lebanon’s grapevine diversity. They also emphasize the importance of considering geographical factors in genetic studies to better understand the dynamics of grapevine distribution and diversity, which are vital for conservation and sustainable cultivation efforts.

After analyzing 20 SSR markers, no matches were found between the genetic profiles of our samples and those from the Armenian database. Altogether, 67 distinct genotypes were identified, with the majority—34 genotypes—not matching any existing profiles in the consulted databases. However, 33 genotypes were successfully matched with known varieties by cross-referencing them with the consulted databases.

These findings underscore the value of employing advanced molecular techniques, as demonstrated in this study, which allow for comparability with global databases and facilitate a comprehensive characterization of grapevine germplasm. Unlike earlier studies conducted in Lebanon [15,45], our approach enabled the identification of local autochthonous cultivars and previously uncharted diversity within the Lebanese grapevine population. Of the 67 genotypes identified, 34 (corresponding to 59 samples) did not match any existing profiles in the consulted databases. Based on collector observations, vernacular names were recorded for the samples: 15 lacked data, 30 were classified as red varieties, 9 were classified as white, and 5 had specific names. These findings indicate a significant number of previously unrecorded grapevine varieties in our collection, suggesting both genetic distinctiveness and previously uncharted diversity.

When compared to the findings of Merheb et al. [52], several Lebanese accessions, such as ‘Cheme’/‘Semaani’, ‘Bayd hamem’, and ‘Al-karm’, aligned with ‘Shami’, ‘Jerdy’, ‘Halabani’ and ‘Halawani’, and ‘Chamouti’, respectively, indicating the presence of autochthonous varieties specific to Lebanon or distinct regions within it. This observation raises critical concerns regarding the preservation and documentation of these cultivars, suggesting they may be under-represented or misidentified in existing genetic repositories. Merheb et al.’s [52] study made a significant contribution by exploring and characterizing underexploited Lebanese grapevine resources, particularly focusing on old or abandoned vines. Using 22 SSR markers, they revealed several cultivars for the first time, including LBN62, identified across various regions under different vernacular names. In comparison, our study identified this genotype as G61, highlighting the complexities of synonyms and homonyms within the dataset.

In fact, our findings revealed significant genotypic variability among accessions initially classified as the same variety based on field observations, highlighting the complexity of grapevine classification. For example, ‘Tfaifihi’ exhibited five distinct genotypes, while both ‘Meksassi’ and ‘Beitamouni’ displayed three each, and ‘Al-busat’ corresponded to two distinct genotypes. This variability underscores the challenges of mislabeling and the potential influence of genetic drift within cultivated varieties. An example of homonymy is the use of ‘Al-busat’ for two genetically distinct varieties, with one sample matching ‘Beitamouni’ and another matching ‘Ganzin 1′. Cases of misnaming were also observed, such as ‘Meksassi’, a distinct variety that was incorrectly matched with ‘Blanc de Dellys’. The analysis further identified several cases of synonymy, where different vernacular names referred to the same genetic variety. For instance, ‘Biyadi’ and ‘Aasimi’ were both matched to ‘Assoued Kere’, while ‘Hifawi’, ‘Zehlewe’, and ‘Maghdouche’ all corresponded to ‘Dabouki’. Similarly, ‘Derble’ and ‘Jouzene’ were synonymous with ‘Maa’tar’, ‘Tefahi’ and ‘Tfaifihi’ matched with ‘Verigo’, and ‘Al-wadi’ corresponded to ‘Zeitouni’. Additionally, ‘Merwah’ was identified as synonymous with ‘Blanc de Dellys’, and other synonymous groupings were observed, such as ‘Cheme’/’Semaani’, linked to ‘Shami’, ‘Jerdy’, and ‘Halabani’, while ‘Bayd hamem’ and ‘Al-karm’ corresponded to ‘Halawani’ and ‘Chamouti’.

While our study identified several grapevine varieties of putative Lebanese origin, including ‘Beitamouni’ and ‘Obeidi’, it notably lacked the cultivars ‘Zeini Abiad’ and ‘Afus Ali’. Despite the widespread documentation of ‘Afus Ali’ in previous studies across the Mediterranean [37,53], its absence raises important questions about its current status in Lebanon. This situation may result from factors such as changing agricultural practices, environmental pressures, or human-mediated selection, highlighting an urgent need for further research into the dynamics affecting the distribution and genetic diversity of these cultivars. The conservation and documentation of these varieties are essential for preserving the region’s viticultural heritage and ensuring sustainable grape-growing practices, as they play a crucial role in maintaining genetic diversity and supporting local agriculture and cultural identity.

### 3.2. Evaluation of Genetic Diversity and Relationships Within Lebanese Grape Germplasm Through Microsatellite Analysis

The statistics concerning the discriminatory efficiency of the 20 SSR markers can be found in Table 1. The substantial count of different alleles (228) serves as evidence of the considerable observed genetic variability. The number of alleles observed at each SSR locus varied, ranging from 5 (for VVIQ52 and VVIN73) to 19 (for VVIV67), with an average of 11.4 alleles per locus. This variation suggests differing degrees of genetic diversity at different loci. The effective number of alleles ranged from 1.343 for the VVIN73 locus to 7.703 for the VVS2 locus, with an average of 4.896. High effective allele numbers were also observed for VVIV67 (7.531) and VVMD5 (6.992) loci. Shannon’s information index (I), which reflects polymorphism, was highest for the VVIV67 locus (2.384) and lowest for VVIN73 (0.579), with an average of 1.774 across all loci.

When assessing genetic variability among the analyzed varieties using microsatellite markers, the observed heterozygosity (Ho) ranged from 0.141 (for VVIN73) to 0.881 (for VMC1B11), with a mean value of 0.732 across all loci. The expected heterozygosity (He) values ranged between 0.255 (for VVIN73) and 0.870 (for VVS2), with an average He of 0.757. The average values for observed and expected heterozygosity were relatively close, with observed heterozygosity at approximately 0.732 and expected heterozygosity at 0.757. The proximity of these values suggests a balance between observed and expected genetic diversity. However, the fact that observed heterozygosity is lower than expected may indicate that the population is not in complete Hardy–Weinberg equilibrium. This deviation could imply the influence of evolutionary forces, such as inbreeding, selection, or population structure, affecting allele frequencies within the population. The fixation index (Fis) varied from −0.06 (for VMC1B11) to 0.449 (for VVIN73), with an average value of 0.046.

Information regarding allele size (AS) and frequencies (AF) for each of the microsatellites can be found in Supplementary Table S5. All of the examined loci exhibited at least one allele with a frequency exceeding 0.20. The VVIN73 alleles had the highest frequency at 0.859, followed by the VVIB01 alleles with a frequency of 0.629.

**Figure 2:**
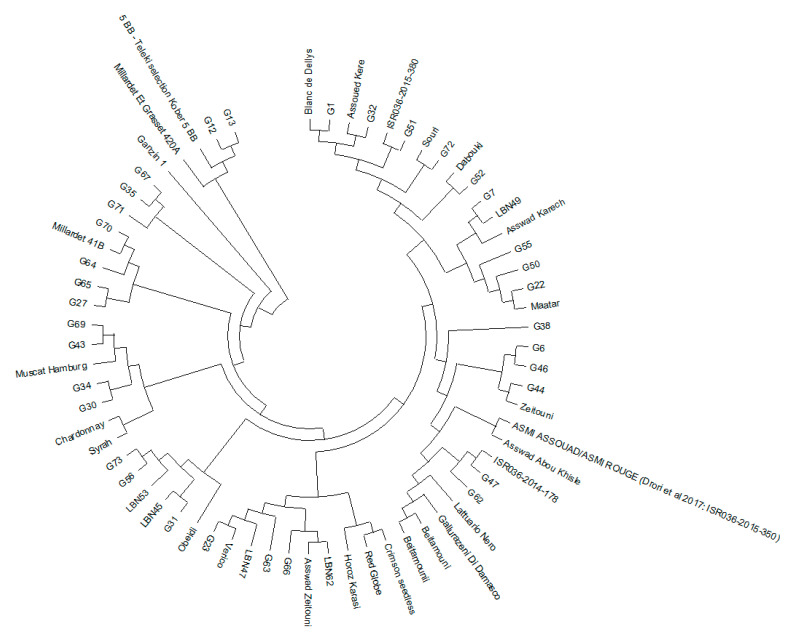
Neighbor-joining dendrogram illustrating the genetic relationships among our 67 genotypes based on 20 SSR loci.

A neighbor-joining (NJ) distance tree was created to investigate the genetic connections among the 67 genotypes by analyzing the allele frequencies of 20 SSR loci. The resulting phylogenetic tree (Figure 2) provides significant insights into the genetic relationships, geographic origins, and potential domestication pathways of the studied varieties. The dendrogram reveals a coherent clustering pattern that aligns with the geographic origins and domestication histories of the varieties. A prominent feature of the dendrogram is the presence of a distinct “external group” comprising rootstock varieties (‘Millardet et Grasset 420A’, ‘Kober 5 BB’, and ‘Ganzin 1′), which are genetically distinct from the other genotypes. These rootstocks likely originate from hybrids involving other species within the *Vitis* genus, explaining their clear separation from traditional cultivars. Additionally, some genotypes (G30, G34, G43, and G69) cluster closely with well-known foreign varieties like ‘Muscat Hamburg’, suggesting a possible shared ancestry or historical genetic exchange. The two commercial varieties, ‘Crimson Seedless’ and ‘Red Globe’, are grouped together in the dendrogram, further emphasizing their shared genetic background. Regional varieties from countries like Turkey, Syria, Tunisia, and Israel form distinct clusters that extend to local genotypes. Notably, some Lebanese samples, specifically G56 and G73, cluster with LBN53, while G31 clusters with LBN45 and closely with ‘Obeidi’—varieties believed to be autochthonous to Lebanon. Further analysis suggests that the isolation of grapevine populations in high-altitude regions may influence their phylogenetic structure (Figure S1). Varieties from high-altitude regions (1600 m), such as G30 and G34 from Bcharre and Ehden, respectively, cluster together. On the other hand, varieties G56 and G73, also from high-altitude areas, form a distinct cluster, suggesting that genetic similarities in these cases might be more reliably linked to altitude and environmental factors.

## 4. Discussion

The domestication of grapevines stands out as a remarkable process in human agricultural innovation and due to its important impact on the viticulture industry. Over time, the cultivation of wild grapevines has evolved, leading to the development of a wide range of grape varieties with distinct characteristics, flavors, and adaptabilities. Archeological evidence suggests that the origins of grapevine domestication can be traced back to the fertile crescent region. Therefore, knowing the historical context and genetic characterization of grapevines is essential for the future of grape breeding, adaptation, and conservation. The objective of the current study was to identify local grapevine varieties in Lebanon, with a specific focus on pinpointing previously unidentified and indigenous genotypes. Understanding the genetic diversity and relationships among Lebanese grapevine varieties is crucial for recognizing distinct gene pools within the grapevine population. The findings of our research have revealed that Lebanese grapevines, collected in various regions of Lebanon, are characterized by a rich mix of various genetic types. These samples were sourced from a variety of settings, including old, abandoned vineyards, home gardens, and vineyards near rivers. Whenever possible, we took care to document the specific names of grapevine varieties for each sample. Additionally, we diligently recorded the geographic coordinates and elevations of all the collected samples. This comprehensive data collection process was undertaken with the intention of creating an extensive dataset that could facilitate a thorough examination of the genetic aspects of Lebanon’s grape varieties.

The accurate identification of local cultivars is the first step towards their conservation. Traditionally, the identification of grape varieties has been based on the morphological features of vegetative and reproductive structures [54]. However, ampelography has certain limitations. An alternative approach could involve starting with genetic characterization, followed by ampelographic studies to map the morphological traits of a given genotype. These data could then be used to train a model that enables the fast identification of cultivars based on morphological features once the model is fully developed. Microsatellites or Simple Sequence Repeat (SSR) markers are highly polymorphic molecular markers and have proven to be effective tools for identifying new or locally adapted grapevine varieties. Natural variations in the length of SSR occur as grapevines grow, primarily due to different types of mutations. These variations are responsible for the genetic diversity within grapevine varieties.

The accurate identification and classification of grapevine varieties are essential for conserving genetic diversity, supporting breeding programs, and ensuring the sustainability of viticulture worldwide. However, traditional naming practices, shaped by cultural and historical contexts, often obscure the genetic relationships between cultivars. This study highlights the complex web of synonymy, homonymy, and misnaming within Lebanon’s grapevine germplasm, emphasizing the limitations of vernacular naming systems and highlighting the need for molecular characterization to resolve these ambiguities.

Our findings clarify how different names may refer to the same cultivar or the same name may refer to distinct cultivars. The reliance on local names, whether for indigenous or imported varieties, creates a complex network of synonyms and homonyms, which reflects cultural and historical influences but also complicates accurate identification and classification. These challenges, exemplified by the misnaming of ‘Meksassi’, underscore the importance of molecular studies for reliable cultivar identification. Addressing these issues is crucial for the accurate characterization, conservation, and sustainable utilization of Lebanon’s diverse grapevine germplasm, and for improving data reliability and communication within the viticulture research community. However, further molecular studies and comprehensive field surveys are necessary to fully validate and confirm these results.

In the context of Lebanese grapevine diversity, despite the historical and economic significance of Lebanese grape cultivars like ‘Beitamouni’ and ‘Obeidi’, which are crucial for table grape and wine production [55], our study revealed only seven samples of ‘Beitamouni’ and three samples of ‘Obeidi’. This can be explained by the fact that our sampling focused on old and/or abandoned grapes. These results suggest potential for undiscovered genetic diversity among Lebanese grape varieties and underscore the need for further research to document and conserve this vital agricultural heritage. The absence of well-known cultivars, along with the discovery of unrecorded varieties, highlights the importance of comprehensive genetic assessments in understanding local viticulture.

The detection of ‘Beitamouni’ and ‘Obeidi’, along with other native varieties identified by Merheb et al. (including LBN45, LBN47, LBN49, LBN53, LBN62, ‘Souri’, and ‘Asmi’), confirms their status as indigenous to the region. The fact that these varieties were detected in two independent studies confirms their status as native. This consistency highlights the importance of documenting grapevine diversity to address challenges in Lebanese viticulture. Conservation strategies are crucial for enhancing the resilience and sustainability of local grape-growing, preserving the region’s unique agricultural heritage.

To contextualize our findings, we identified five samples (G15 and G26) that exhibited genetic similarities to accessions reported by Drori et al. [56]. This suggests potential uniqueness in the grapevine varieties examined, indicating a close genetic relationship between our samples and those documented. This genetic relationship may arise from local adaptation, environmental influences, or human-mediated selection practices, reflecting distinct evolutionary pathways or breeding histories. Given the geographic proximity of Lebanon and Israel, this may highlight the unique viticultural practices and ecological conditions that have shaped grapevine development over time. This genetic diversity underscores the complex interplay of historical trade routes, cultural practices, and natural selection that has shaped viticulture in the Eastern Mediterranean. Our findings align with historical accounts suggesting that the region has served as a crossroads for grape cultivation, with the Phoenicians playing a pivotal role in spreading viticulture throughout the Eastern Mediterranean. Furthermore, the results obtained by Merheb et al. [52] confirm these observations and may provide insights into the genetic resilience and adaptability of these varieties in response to changing conditions.

Interestingly, one sample (G21) closely matched ‘Beitamouni’ but showed genetic mismatches, suggesting a close relationship with notable divergence, possibly due to autofertilization. While grapevines typically outcross, self-fertilization can occur due to environmental factors, genetic predisposition, or vine architecture. These mismatches could reflect the effects of inbreeding, where traits specific to ‘Beitamouni’ are retained while other variations are lost or altered. They may also indicate adaptation to different environmental conditions, leading to changes that enhance viability and reproductive success. Understanding self-fertilization dynamics is crucial for breeding and conservation, as it influences genetic variability and impacts efforts to improve disease resistance, fruit quality, and environmental adaptation.

In terms of broader genetic profiles, the remaining 33 genotypes, corresponding to 143 collected samples, were successfully associated with known grapevine varieties, including notable wine, table, and rootstock varieties, through cross-referencing with consulted databases. Notably, ‘Blanc De Dellys’ (29 samples) and ‘Dabouki’ (27 samples) emerged as the most frequent varieties, prevalent in neighboring Tunisia and Israel, respectively. This finding indicates a shared cultural and historical bond among these grape varieties that transcends borders. It highlights the historical connection to Carthage, a Phoenician colony located in modern-day Tunisia, founded by settlers from Tyre in around 814 BCE. As a key player in the dissemination of agricultural practices, including viticulture (grape cultivation), Carthage significantly influenced the Mediterranean region [7].

Furthermore, our study identified a variety of foreign grape cultivars, including ‘Muscat Hamburg’, ‘Crimson’, ‘Syrah’, ‘Red Globe’, ‘Lattuario Nero’, and ‘Chardonnay’, alongside notable rootstocks such as ‘Millardet et Grasset 420A’, ‘Kober 5 BB’, and ‘Ganzin 1′. This finding supports historical accounts of Lebanon’s role as a central hub for grapevine exchange and international breeding. The presence of these rootstocks is particularly significant for two reasons: they thrive in challenging conditions, especially in abandoned plots where traditional varieties may have failed, and their resilience enhances viticulture sustainability. Rootstocks improve grapevine resilience to environmental stresses like drought and disease, thanks to their genetic diversity and ability to adapt to various soils and climates. Moreover, these rootstocks reflect a long-standing viticultural legacy in Lebanon, where they have been relied upon for generations. While they may not exhibit the varietal typicity of newer cultivars, they are essential for maintaining vineyard health and productivity. Their integration with commercial varieties has led to the development of more resilient and adaptable cultivars, benefiting local farmers and the broader industry. Varieties like ‘Red Globe’ and ‘Crimson Seedless’ exemplify this trend, incorporating traits such as enhanced disease resistance, climate adaptability, and improved fruit quality.

This genetic exchange not only diversifies Lebanon’s grape production but also strengthens its agricultural economy by meeting market demand for high-quality table grapes. The successful integration of these foreign varieties underscores the importance of genetic diversity in viticulture, enabling growers to adapt to environmental challenges and shifting consumer preferences. Overall, these developments highlight the dynamic nature of grape breeding and its critical role in ensuring the sustainability of Lebanon’s grape industry.

Building on Lebanon’s rich viticultural legacy—particularly in the Bekaa Valley, which boasts a favorable climate and fertile soils—the country has developed a well-established wine industry that traces its roots back to the Phoenicians. Renowned for their winemaking expertise, the Phoenicians significantly influenced viticulture across the Mediterranean as ancient seafarers and traders. They established vineyards and developed winemaking techniques that impacted regions such as modern-day Armenia. Their extensive trade routes facilitated the exchange of grape cultivation practices and winemaking expertise, contributing to the rich viticultural traditions in the Armenian Highlands [57].

Interestingly, there are no matches in the Armenian database when comparing the profiles of our samples. This absence may result from several factors: the database might primarily focus on Armenian viticulture, potentially overlooking broader historical influences from Lebanon. Additionally, many ancient exchanges may not have been thoroughly documented in databases that concentrate on specific cultural contexts. Ultimately, the lack of matches underscores the need for more comprehensive research and documentation to capture the extent of cultural contributions to Lebanese viticulture.

Identifying the origins of grapevine cultivars, particularly their indigenous status, is complex and requires integrating molecular data with the existing literature and ampelographic records. Such research uncovers the detailed history and migration of grapevines, suggesting that these cultivars were extensively shared across regions rather than confined to a single country. Furthermore, it emphasizes the enduring value and selection of local varieties by communities, which played a crucial role in their cultivation and preservation.

In terms of allelic diversity, our study identified 228 alleles, indicating substantial allelic diversity within the Lebanese grapevine population. This result is consistent with a previous study in Lebanon, which reported 232 alleles were found [52], suggesting shared genetic influences. However, the allelic diversity in our study is lower than that reported in other regions, such as Armenia, which documented 347 alleles [47], and Spain, where 261 alleles were identified [58]. Conversely, Cyprus displayed lower diversity, with only 67 alleles detected from 11 SSR markers [42]. Additionally, a broader Mediterranean sample revealed 158 alleles [59]. Furthermore, two recent studies from Turkey provided additional insights into regional diversity. Arslan et al. [60] documented 196 alleles in Anatolian Kara Grapevine, while Özmen et al. [61] identified 141 alleles among Aegean region cultivars.

Although our study highlights noteworthy allelic diversity, the 228 alleles identified may be lower than those reported elsewhere due to factors such as geographic and environmental influences, the number of SSR markers used, and the genetic background of the sampled grapevines. Additionally, historical cultivation practices and selective breeding may have contributed to reduced genetic variation in our area, emphasizing the complex interplay between intrinsic and extrinsic factors shaping allelic diversity.

In terms of genetic diversity, the average expected heterozygosity (He) of 0.757 in our study is consistent with values reported in other Mediterranean regions, such as those by Merheb et al. (He = 0.760) [52] and Hizarci et al. (He = 0.759) [62]. However, Margaryan et al. (He = 0.789) [47] and Štajner et al. (He = 0.775) [63] reported slightly higher values, suggesting these populations may possess greater genetic variation. Lower heterozygosity values were reported by Arslan et al. (He = 0.746) [60] and Özmen et al. (He = 0.725) [61] in Turkey.

Fis values ranged from −0.06 (for VMC1B11) to 0.449 (for VVIN73), with an average of 0.046, suggesting a low level of inbreeding, although the positive Fis value indicates a slight excess of homozygotes relative to expectations under Hardy–Weinberg equilibrium. While this suggests some degree of inbreeding, its magnitude is not substantial, implying that the population retains a relatively healthy level of genetic diversity. This low average Fis value is beneficial for maintaining genetic variability, which is crucial for the population’s adaptability to environmental changes and challenges. Overall, the findings indicate that the population is not significantly impacted by inbreeding, supporting its long-term genetic health. Continued monitoring and analysis will be essential to track trends in genetic diversity and population structure over time.

Additionally, the examination of various loci revealed that each contained at least one allele with a frequency exceeding 0.20, indicating the relatively common presence of these alleles in the population. Notably, two loci stood out due to their high-frequency alleles: the VVIN73 locus at 263 bp exhibited the highest frequency at 0.859, indicating a substantial level of genetic uniformity for that specific allele within the population. Similarly, the VVIB01 locus at 290 bp demonstrated a high frequency of 0.629, suggesting a significant presence of this allele as well. These findings suggest that these specific alleles are both prevalent and widespread within the population, thereby contributing to its genetic diversity. These loci contribute significantly to the genetic structure of the population and are consistent with findings by Ghrissi et al. (0.862 for VVIN73) [58] and Margaryan et al. (0.661 for VVIN73). Notably, our frequency for the VVIB01 locus (0.629) is higher than the value of 0.423 reported by Margaryan et al. [47], which may reflect potential regional differences or varying selective pressures across different grapevine populations.

In addition, allele frequencies for loci VrZAG67, VVIH54, VVIN16, VVIP60, VVIQ52, VVMD24, VVMD7, VVMD27, VVMD28, VVMD32, and VrZAG83 ranged from 0.3 to 0.46. Detailed frequency ranges for all 20 markers are provided in Supplementary Table S5.

Overall, our findings highlight the higher prevalence of the VVIN73 and VVIB01 alleles, suggesting that environmental factors or genetic drift may influence allele distribution across grapevine populations. While this study identifies specific high-frequency alleles, the broader literature reveals a complex landscape of allele frequencies shaped by ecological and evolutionary dynamics. Understanding these variations is crucial for future conservation efforts to preserve genetic diversity, particularly in Lebanese grapevine varieties, where such diversity is reflected in phylogenetic relationships.

The clustering of grape varieties by region—such as European versus Middle Eastern groups—highlights the impact of local breeding practices on grapevine genetics. For example, despite differences in color and wine type, French varieties like ‘Syrah’ and ‘Chardonnay’ cluster together, reflecting their shared lineage and regional selection history. This pattern underscores how centuries of selective breeding aimed at enhancing traits like yield and resilience have shaped grape diversity. A distinct feature of the dendrogram is the clustering of rootstock varieties, which illustrates their genetic divergence from traditional cultivars. In addition, the proximity of genotypes like G12 and G13 to this group may indicate that they are descendants of these rootstocks or wild populations once cultivated for propagation. Further genetic analysis could clarify whether these closely associated genotypes are relics of earlier domestication efforts or remnants of wild populations. Additionally, the clustering of genotypes like G30, G34, G43, and G69 with ‘Muscat Hamburg’ suggests a shared ancestry or historical genetic exchange. Known for its aromatic qualities and dual use as table grapes and wine, ‘Muscat Hamburg’ may have contributed traits such as flavor and adaptability to local varieties through crossbreeding. This clustering reflects the influence of foreign varieties introduced through trade, enriching the genetic diversity of local grapes. These connections underscore the complex history of grapevine domestication, where both intentional breeding and unintentional hybridization have shaped local populations. Further research could reveal whether these relationships stem from recent or ancient exchanges. The grouping of ‘Crimson Seedless’ and ‘Red Globe’ together underscores their shared ancestor, Sultanina—a historically significant variety with uncertain origins, possibly tracing back to Central Asia or the Greece–Turkey region, despite both being developed in the United States. This link emphasizes the historical movement of grapevine genetic material across regions. Furthermore, the proximity of ‘Horoz Karasi’, a red grape indigenous to Turkey, to these internationally recognized varieties suggests that trade routes and migration facilitated the genetic exchange of grapevine varieties. This supports the idea that geographic origin plays a significant role in the genetic grouping observed, and highlights the influence of regional lineage in shaping genetic relationships. Regional varieties from countries like Turkey, Syria, Tunisia, and Israel form distinct clusters that extend to local genotypes. For instance, varieties like ‘Asswad Abou Khisle’ and ‘Asmi’, from the Middle Eastern region, cluster together, suggesting a shared genetic background shaped by regional domestication practices. These varieties, likely originating in Syria or Lebanon, are genetically similar to local samples, indicating either common ancestry or a history of genetic exchange possibly facilitated by ancient trade routes. These connections suggest that regional grape varieties may have influenced each other through deliberate crossbreeding or unintentional hybridization as vines were transported along historical pathways. The clustering of ‘Lattuario Nero’ with ‘Beitamouni’ raises questions about the historical origins of certain varieties. ‘Lattuario Nero’, traditionally associated with Italy and known by synonyms such as ‘Jerusalem grape’, may have ancient connections to trade routes established by Phoenician merchants, indicating its spread across regions via early trade. This observation aligns with theories proposing the spread of grapevine varieties across the Mediterranean and beyond through ancient trading networks.

The clustering of Lebanese samples, specifically G56 and G73 with LBN53 and G31 with LBN45 and ‘Obeidi’, reinforces the idea that these samples are locally adapted, underscoring the distinct profile of native Lebanese varieties. This highlights the importance of regional adaptation and preservation, especially in areas with such unique varietal histories. Further analysis suggests that geographical isolation and environmental factors—such as altitude—may influence the phylogenetic structure of grapevines in these regions. Varieties from high-altitude regions (1600 m), such as G30 and G34 from Bcharre and Ehden, cluster together, which may indicate that altitude and environmental factors are shaping their genetic characteristics. However, due to the low frequency of these varieties (with only one sample each), it is difficult to confidently attribute their clustering to altitude alone. These varieties may represent recently sprouted seeds rather than established, old varieties. The distinct cluster of varieties G56 (six samples) and G73 (two samples), which also come from high-altitude areas, further supports the possibility that altitude and environmental factors may play a role in shaping genetic diversity. Farmers in mountainous areas may have maintained certain varieties through selective breeding, while varieties from lower regions might have struggled to adapt to these specific conditions. However, more samples and further analysis are needed to draw definitive conclusions about the role of altitude in shaping grapevine genetic structure.

Overall, the analysis of the dendrogram reveals the complexity of grapevine phylogenetics, emphasizing the influence of regional domestication practices, the historical movement of genetic lineages, and environmental factors. The genetic relationships depicted in this tree underscore the rich diversity of Lebanese grapevines and the various factors that contribute to their phylogenetic structure. Further investigation into the genetic makeup and historical background of these varieties could deepen our understanding of grapevine evolution and contribute to ongoing conservation and breeding efforts.

This complexity in genetic diversity parallels the growing understanding of the Phoenician influence on viticulture in the Western Mediterranean [64]. Archeological evidence, including the presence of wine presses [65], trench agricultural systems [66], and the archaeobotanical record [67,68,69,70], indicates that the Phoenicians played a significant role in developing grape cultivation. Additionally, findings of *V. vinifera* alongside animal remains [71,72] suggest that winemaking practices were integrated into their lifestyle, utilizing grape waste for meat preservation due to its antioxidant properties [73]. This historical context further enriches our understanding of the genetic diversity observed in contemporary grape populations, highlighting the deep-rooted connections between genetic variability and cultural practices in viticulture.

## 5. Conclusions

The investigation of Lebanon’s traditional grape-growing regions has revealed significant genetic diversity among grapevines, using microsatellite markers to identify both known and previously unrecorded varieties. This research highlights the presence of distinctive and potentially rare grapevine types that had not been documented before, providing insights into evolutionary processes and conservation strategies essential for preserving viticultural heritage. To further enhance grapevine identification, a reverse approach could be adopted, starting with genetic characterization followed by ampelographic studies of different genotypes to create a genotype/morphological reference. These data could then be used to develop a model for the rapid identification of cultivars based on their morphological features once the model is fully established. Combining genetic analyses with ampelographic studies is essential for defining and conserving this unique genetic heritage, while continued research and collaboration are vital for discovering and protecting additional distinctive grapevine varieties.

Looking ahead, future research into grape domestication and distribution could benefit from expanding sampling to less-studied regions, revealing new grape varieties and historical connections between grape-growing areas. Collaborating with historians, archeologists, and geneticists will provide a deeper understanding of the cultural and historical context of grape cultivation. Technological advancements, such as whole-genome sequencing and metabolomics, will enhance the identification of genetic diversity. Additionally, investigating the effects of climate change on grapevine genetics and distribution will be crucial for adapting viticulture to future challenges. These efforts will contribute to preserving and sustainably managing viticultural resources in the eastern Mediterranean.

In summary, these findings enhance our understanding of grapevine genetic diversity and highlight the critical need to conserve both local and international genetic resources for future agricultural and research initiatives. Such efforts will support the sustainability of viticulture, particularly in the face of climate change and emerging pathogens.

## Figures and Tables

**Table 1:** Descriptive statistics and genetic diversity of the 67 genotypes across the 20 microsatellite loci.

Locus	Ra (bp)	Na	Ne	I	Ho	He	Fis	N
VVIN16	149–159	6	3.139	1.337	0.688	0.681	−0.009	64
VVIV67	326–391	19	7.531	2.384	0.800	0.867	0.078	65
VVMD27	175–215	14	4.336	1.793	0.723	0.769	0.060	65
VVMD28	218–274	16	4.949	1.983	0.776	0.798	0.027	67
VVMD32	238–272	10	5.328	1.939	0.841	0.812	−0.036	63
VVMD5	221–264	14	6.992	2.134	0.833	0.857	0.028	66
VrZAG67	124–176	15	5.599	2.041	0.848	0.821	−0.033	66
VMC1B11	167–194	13	5.922	2.006	0.881	0.831	−0.060	67
VVIB01	284–307	9	2.122	1.060	0.530	0.529	−0.003	66
VVIH54	140–181	14	5.592	2.075	0.636	0.821	0.225	66
VVIN73	254–267	5	1.343	0.579	0.141	0.255	0.449	64
VVIP60	306–333	12	4.506	1.762	0.797	0.778	−0.024	64
VVIQ52	82–93	5	3.432	1.315	0.723	0.709	−0.020	65
VVMD24	202–222	8	4.950	1.793	0.803	0.798	−0.006	66
VVMD25	235–269	11	5.673	1.875	0.781	0.824	0.052	64
VVMD7	229–263	13	5.351	1.948	0.803	0.813	0.012	66
VVS2	125–155	12	7.703	2.184	0.785	0.870	0.098	65
VrZAG83	166–201	9	3.344	1.467	0.672	0.701	0.041	64
VrZAG62	173–219	13	5.139	1.967	0.851	0.805	−0.056	67
VrZAG79	239–263	10	4.964	1.842	0.727	0.799	0.089	66
Total		228						
Min		5	1.343	0.579	0.141	0.255	−0.060	63
Max		19	7.703	2.384	0.881	0.870	0.449	67
Mean		11.4	4.896	1.774	0.732	0.757	0.046	

Ra, range of allele size (bp); Na, number of different alleles; Ne, effective alleles; I, Shannon’s information index; Ho, observed heterozygosity; He, expected heterozygosity; Fis, fixation index.

## Data Availability

All data generated in this study will be added to national and international databases. Molecular data are available in Supplementary Table S3.

## Supplementary Materials

The following supporting information can be downloaded at https://www.mdpi.com/article/10.3390/genes15121617/s1: Figure S1: Geographic distribution of sampling sites in Lebanon based on altitude in meters (left) and precipitation in mm/year (right); Table S1: Summary of data for the 202 collected grapevine samples; Table S2: Primer sequences, chromosome positions, span, fluorophore, PCR multiplexing, and the reference utilized within the study; Table S3. Microsatellite profiles for 20 nSSR loci of the non-redundant genotypes analyzed; Table S4: Genetic matches with VIVC database and identification of non-redundant genotypes; Table S5: Allele size (AS, bp) and allele frequency (AF) for the 20 nSSR marker. [74,75,76,77,78]

## References

[1] McGovern P., Jalabadze M., Batiuk S., Callahan M.P., Smith K.E., Hall G.R., Kvavadze E., Maghradze D., Rusishvili N., Bouby L. (2017). Early Neolithic Wine of Georgia in the South Caucasus. Proc. Natl. Acad. Sci. USA.

[2] Mariani L., Cola G., Maghradze D., Failla O., Zavatti F. (2018). Influence of Climate Cycles on Grapevine Domestication and Ancient Migrations in Eurasia. Sci. Total Environ..

[3] Terral J.-F., Tabard E., Bouby L., Ivorra S., Pastor T., Figueiral I., Picq S., Chevance J.-B., Jung C., Fabre L. (2010). Evolution and History of Grapevine (*Vitis vinifera*) under Domestication: New Morphometric Perspectives to Understand Seed Domestication Syndrome and Reveal Origins of Ancient European Cultivars. Ann. Bot..

[4] Capdevila R.B. (1997). Presence of “*Olea europaea*” and “*Vitis vinifera*” in Archaeological Sites from the Iberian Peninsula. Lagascalia.

[5] Adler D., Tushabramishvili N. (2004). Middle Palaeolithic Patterns of Settlement and Subsistence in the Southern Caucasus. Settlement Dynamics of the Middle Paleolithic and Middle Stone Age.

[6] Miller N.F. (2008). Sweeter than Wine? The Use of the Grape in Early Western Asia. Antiquity.

[7] Harutyunyan M., Malfeito-Ferreira M. (2022). The Rise of Wine among Ancient Civilizations across the Mediterranean Basin. Heritage.

[8] Iriarte-Chiapusso M.J., Ocete-Pérez C.A., Hernández-Beloqui B., Ocete-Rubio R. (2017). *Vitis vinifera* in the Iberian Peninsula: A Review. Plant Biosyst. Int. J. Deal. Asp. Plant Biol..

[9] International Organisation of Vine and Wine (OIV) (2017). Annual Report on the State of the Vitivinicultural Sector.

[10] Harfouche R. (2012). Aux Origines de La Viticulture Méditerranéenne: Le Vignoble Du Mont Liban. L’univers du Vin: Hommes, Paysages et Territoires.

[11] Zohary D. (1996). The Domestication of the Grapevine *Vitis vinifera* L. in the Near East. The Origins and Ancient History of Wine.

[12] Orsingher A., Kamlah J., Sader H., Schmitt A., Amicone S., Berthold C. (2021). Making, Trading and Consuming Phoenician Wine. The Ancient Near East Today. Current News About the Ancient Past IX.9. https://www.asor.org/anetoday/2021/09/.

[13] Wicenciak U. (2019). Aspects of Economic Activity in Phoenicia during Roman and Byzantine Times. The Case of Olive Oil and Amphora Production in Chhim, in the *Chora* of Sidon. Levant.

[14] Tabaja N. Current Status of Registered Fruit Tree Seedlings in Lebanon and Potential Impact on Germplasm Diversity. Proceedings of the III International Symposium on Horticulture in Europe-SHE2016.

[15] Chalak L., Touma S., Rahme S., Azzi R., Guiberteau F., Touma J.-A. (2016). Assessment of the Lebanese Grapevine Germplasm Reveals a Substantial Diversity and a High Potential for Selection. Bio. Web Conf..

[16] Markou M., Kavazis A. Agricultural Situation Report of Cyprus and the Market and Trade Policies for Fruit/Vegetable and Olive Oil. Proceedings of the 98th EAAE Seminar ‘Marketing Dynamics within the Global Trading System: New Perspectives’.

[17] Corrieri F., Piras F., Abou Assi M., Focacci M., Conti L. (2022). Terraced Landscapes of the Shouf Biosphere Reserve (Lebanon): Analysis of Geomorphological Variables. Biodivers. Conserv..

[18] Galet P. (1998). Grape Varieties and Rootstock Varieties.

[19] Hellman E. (2003). Grapevine Structure and Function. Oregon Viticulture.

[20] Tandonnet J.-P., Cookson S.J., Vivin P., Ollat N. (2009). Scion Genotype Controls Biomass Allocation and Root Development in Grafted Grapevine: Scion/Rootstock Interactions in Grapevine. Aust. J. Grape Wine Res..

[21] Keller M., Mills L.J., Harbertson J.F. (2012). Rootstock Effects on Deficit-Irrigated Winegrapes in a Dry Climate: Vigor, Yield Formation, and Fruit Ripening. Am. J. Enol. Vitic..

[22] Dallakyan M., Esoyan S., Gasparyan B., Smith A., Hovhannisyan N. (2020). Genetic Diversity and Traditional Uses of Aboriginal Grape (*Vitis vinifera* L.) Varieties from the Main Viticultural Regions of Armenia. Genet. Resour. Crop Evol..

[23] Pachauri R.K., Meyer L.A., Core Writing Team (2014). Climate Change 2014: Synthesis Report. Contribution of Working Groups I, II and III to the Fifth Assessment Report of the Intergovern-mental Panel on Climate Change.

[24] MedECC (2019). Risks Associated to Climate and Environmental Changes in the Mediterranean Region; Union for the Mediterranean, Plan Bleu. Climate-ADAPT.

[25] Sancho-Galán P., Amores-Arrocha A., Palacios V., Jiménez-Cantizano A. (2020). Identification and Characterization of White Grape Varieties Autochthonous of a Warm Climate Region (Andalusia, Spain). Agronomy.

[26] Lobell D.B., Schlenker W., Costa-Roberts J. (2011). Climate Trends and Global Crop Production Since 1980. Science.

[27] Lipper L., Thornton P., Campbell B.M., Baedeker T., Braimoh A., Bwalya M., Caron P., Cattaneo A., Garrity D., Henry K. (2014). Climate-Smart Agriculture for Food Security. Nat. Clim. Chang..

[28] Jiménez-Cantizano A., Muñoz-Martín A., Amores-Arrocha A., Sancho-Galán P., Palacios V. (2020). Identification of Red Grapevine Cultivars (*Vitis vinifera* L.) Preserved in Ancient Vineyards in Axarquia (Andalusia, Spain). Plants.

[29] Newton S.K., Gilinsky A., Jordan D. (2015). Differentiation Strategies and Winery Financial Performance: An Empirical Investigation. Wine Econ. Policy.

[30] Vikram P., Franco J., Burgueño-Ferreira J., Li H., Sehgal D., Saint Pierre C., Ortiz C., Sneller C., Tattaris M., Guzman C. (2016). Unlocking the Genetic Diversity of Creole Wheats. Sci. Rep..

[31] Pastor i Batalla I., Rovira i Grau J.M. (2013). La investigación arqueológica en los procesos de recuperación de variedades de uva autoctonas tradicionales. Patrimonio Cultural de la Vid y el Vino: Conferencia Internacional.

[32] Emanuelli F., Lorenzi S., Grzeskowiak L., Catalano V., Stefanini M., Troggio M., Myles S., Martinez-Zapater J.M., Zyprian E., Moreira F.M. (2013). Genetic Diversity and Population Structure Assessed by SSR and SNP Markers in a Large Germplasm Collection of Grape. BMC Plant Biol..

[33] Tympakianakis S., Trantas E., Avramidou E.V., Ververidis F. (2023). *Vitis vinifera* Genotyping Toolbox to Highlight Diversity and Germplasm Identification. Front. Plant Sci..

[34] Augusto D., Ibáñez J., Pinto-Sintra A.L., Falco V., Leal F., Martínez-Zapater J.M., Oliveira A.A., Castro I. (2021). Grapevine Diversity and Genetic Relationships in Northeast Portugal Old Vineyards. Plants.

[35] Tsivelikas A.L., Avramidou E.V., Ralli P.E., Ganopoulos I.V., Moysiadis T., Kapazoglou A., Aravanopoulos F.A., Doulis A.G. (2022). Genetic Diversity of Greek Grapevine (*Vitis vinifera* L.) Cultivars Using Ampelographic and Microsatellite Markers. Plant Genet. Resour. Charact. Util..

[36] González R., Vargas A.M., Garnatje T., Vallès J., de Andrés M.T. (2023). Exploring Diversity among Grapevines Varieties (*Vitis vinifera* L.) in Ibiza and Formentera (Balearic Islands, Spain) Using Microsatellite Markers, Ampelographic Methods and an Ethnobotanical Approach. Horticulturae.

[37] Maraš V., Tello J., Gazivoda A., Mugoša M., Perišić M., Raičević J., Štajner N., Ocete R., Božović V., Popović T. (2020). Population Genetic Analysis in Old Montenegrin Vineyards Reveals Ancient Ways Currently Active to Generate Diversity in *Vitis vinifera*. Sci. Rep..

[38] Stavrakaki M., Biniari K. (2017). Ampelographic and Genetic Characterization of Grapevine Varieties (*Vitis vinifera* L.) of the ‘Mavroudia’ Group Cultivated in Greece. Not. Bot. Horti Agrobot. Cluj-Napoca.

[39] Ibáñez J., Vargas A.M., Palancar M., Borrego J., de Andrés M.T. (2009). Genetic Relationships among Table-Grape Varieties. Am. J. Enol. Vitic..

[40] Lacombe T., Boursiquot J.-M., Laucou V., Di Vecchi-Staraz M., Péros J.-P., This P. (2013). Large-Scale Parentage Analysis in an Extended Set of Grapevine Cultivars (*Vitis vinifera* L.). TAG Theor. Appl. Genet. Theor. Angew. Genet..

[41] D’Onofrio C., Tumino G., Gardiman M., Crespan M., Bignami C., de Palma L., Barbagallo M.G., Muganu M., Morcia C., Novello V. (2021). Parentage Atlas of Italian Grapevine Varieties as Inferred From SNP Genotyping. Front. Plant Sci..

[42] Hvarlena T.D., Hadjinicoli A., Atanassov I.I., Ioannou N. (2005). Genotyping *Vitis vinifera* L. Cultivars of Cyprus by Microsatellite Analysis. Vitis J. Grapevine Res..

[43] Giannetto S., Caruana R., La Notte P., Costacurta A., Crespan M. (2010). A Survey of Maltese Grapevine Germplasm Using SSR Markers. Am. J. Enol. Vitic..

[44] De Mattia F., Imazio S., Grassi F., Lovicu G., Tardáguila J., Failla O., Maitt C., Scienza A., Labra M. (2007). Genetic Characterization of Sardinia Grapevine Cultivars by SSR Markers Analysis. OENO One.

[45] Chehade A., Chalak L., Merheb J., Elbitar A., Rmeily E., Madi N., Massaad M. (2022). Genetic and Ampelographic Characterization of Grapevine Accessions Maintained in the Lebanese National Collection. Adv. Hortic. Sci..

[46] Chalak L., Merheb J., Massaad M. (2023). Phenotyping Grapevine Landraces Diversity in the Dry Bekaa Region of Lebanon. Acta Hortic..

[47] Margaryan K., Melyan G., Röckel F., Töpfer R., Maul E. (2021). Genetic Diversity of Armenian Grapevine (*Vitis vinifera* L.) Germplasm: Molecular Characterization and Parentage Analysis. Biology.

[48] This P., Jung A., Boccacci P., Borrego J., Botta R., Costantini L., Crespan M., Dangl G.S., Eisenheld C., Ferreira-Monteiro F. (2004). Development of a Standard Set of Microsatellite Reference Alleles for Identification of Grape Cultivars. Theor. Appl. Genet..

[49] Peakall R., Smouse P.E. (2006). GENALEX 6: Genetic Analysis in Excel. Population Genetic Software for Teaching and Research. Mol. Ecol. Notes.

[50] Nei M. (1973). Analysis of Gene Diversity in Subdivided Populations. Proc. Natl. Acad. Sci. USA.

[51] Tamura K., Stecher G., Kumar S. (2021). MEGA11: Molecular Evolutionary Genetics Analysis Version 11. Mol. Biol. Evol..

[52] Merheb J., Chalak L., Roux C., Laucou V., Ouaini N., Beyrouthy M., Touma J.-A., Lacombe T., This P. (2024). Exploring the Genetic Diversity of Lebanon’s Underexploited Grapevine Resources. Genet. Resour. Crop Evol..

[53] Ghaffari S., Hasnaoui N., Zinelabidine L.H., Ferchichi A., Martínez-Zapater J.M., Ibáñez J. (2013). Genetic Identification and Origin of Grapevine Cultivars (*Vitis vinifera* L.) in Tunisia. Am. J. Enol. Vitic..

[54] Boursiquot J.M.T., Patrice P. (1996). Les Nouvelles Techniques Utilisées En Ampélographie: Informatique et Marquage. J. Int. Sci. Vigne Vin..

[55] Hamie N., Nacouzi D., Choker M., Salameh M., Darwiche L., El Kayal W. (2023). Maturity Assessment of Different Table Grape Cultivars Grown at Six Different Altitudes in Lebanon. Plants.

[56] Drori E., Rahimi O., Marrano A., Henig Y., Brauner H., Salmon-Divon M., Netzer Y., Prazzoli M.L., Stanevsky M., Failla O. (2017). Collection and Characterization of Grapevine Genetic Resources (*Vitis vinifera*) in the Holy Land, towards the Renewal of Ancient Winemaking Practices. Sci. Rep..

[57] Palanjyan R. (2000). Viniculture in Ancient Armenia. Prir. Nat..

[58] Ghrissi H., De Andrés M.T., Andreu L.J., Gogorcena Y. (2022). Genetic Diversity and Structure in a Spanish Grape Germplasm Collection Assessed by SSR Markers. Aust. J. Grape Wine Res..

[59] Ziane L., Bentchikou M., Bravo G., Cabello F., Martínez-Zapater J. (2009). Molecular Identification and Genetic Relationships of Algerian Grapevine Cultivars Maintained at the Germplasm Collection of Skikda (Algeria). Vitis Geilweilerhof.

[60] Arslan N., Yılmaz Baydu F., Hazrati N., Yüksel Özmen C., Ergönül O., Uysal T., Yaşasın A.S., Özer C., Boz Y., Kuleyin Y.S. (2023). Genetic Diversity and Population Structure Analysis of Anatolian Kara Grapevine (*Vitis vinifera* L.) Germplasm Using Simple Sequence Repeats. Horticulturae.

[61] Yüksel Özmen C., Baydu F., Hazrati N., Uysal T., Yaşasin A.S., Özer C., Büyük B.P., Boz Y., Özünlü B., Ergül A. (2023). Genetic Diversity and Population Structure Analysis of Grapevine Germplasm of the Aegean Region (Türkiye) by SSR Markers. Turk. J. Agric. For..

[62] Hizarci Y., Ercişli S., Yuksel C., Ergül A. (2013). Genetic Characterization and Relatedness among Autochthonous Grapevine Cultivars from Northeast Turkey by Simple Sequence Repeats (SSR). J. Appl. Bot. Food Qual..

[63] Štajner N., Tomić L., Ivanišević D., Korać N., Cvetković-Jovanović T., Beleski K., Angelova E., Maraš V., Javornik B. (2014). Microsatellite Inferred Genetic Diversity and Structure of Western Balkan Grapevines (*Vitis vinifera* L.). Tree Genet. Genomes.

[64] Botto M. (2013). The Phoenicians and the Spread of Wine in the Central West Mediterranean. Patrimonio Cultural de la Vid y el Vino: Conferencia Internacional.

[65] Gómez Bellard C., Guérin P., Pérez Jordà G. (1993). Témoignage d’une Production de Vin Dans l’Espagne Préromaine. Bull. Corresp. Hell..

[66] Echevarría A., Vera-Rodríguez J.C. (2013). Sistemas agrícolas del i milenio A.C. en el yacimiento de la orden-seminario de huelva. viticultura protohistórica a partir del análisis arqueológico de las huellas de cultivo. Patrimonio Cultural de la Vid y el Vino: Conferencia Internacional.

[67] Buxó R. (2008). The Agricultural Consequences of Colonial Contacts on the Iberian Peninsula in the First Millennium b.c. Veg. Hist. Archaeobotany.

[68] Pérez-Jordà G., Peña-Chocarro L., García Fernández M., Vera Rodríguez J.C. (2017). The Beginnings of Fruit Tree Cultivation in the Iberian Peninsula: Plant Remains from the City of Huelva (Southern Spain). Veg. Hist. Archaeobotany.

[69] Pérez-Jordà G., Peña-Chocarro L., Pardo-Gordó S. (2021). Fruits Arriving to the West. Introduction of Cultivated Fruits in the Iberian Peninsula. J. Archaeol. Sci. Rep..

[70] Ucchesu M., Orrù M., Grillo O., Venora G., Usai A., Serreli P.F., Bacchetta G. (2015). Earliest Evidence of a Primitive Cultivar of *Vitis vinifera* L. during the Bronze Age in Sardinia (Italy). Veg. Hist. Archaeobotany.

[71] Moricca C., Bouby L., Bonhomme V., Ivorra S., Pérez-Jordà G., Nigro L., Spagnoli F., Peña-Chocarro L., van Dommelen P., Sadori L. (2021). Grapes and Vines of the Phoenicians: Morphometric Analyses of Pips from Modern Varieties and Iron Age Archaeological Sites in the Western Mediterranean. J. Archaeol. Sci. Rep..

[72] Portas L., Farina V., Vais C.D., Carcupino M., Gazza F., Sanna I., Zedda M. (2015). Anatomical Study of Animal Remains from Phoenician-Punic Amphorae Found in the Santa Giusta Pond, Sardinia (Italy). J. Biol. Res. Boll. Soc. Ital. Biol. Sper..

[73] Sabato D., Peña-Chocarro L., Ucchesu M., Sarigu M., Del Vais C., Sanna I., Bacchetta G. (2019). New Insights about Economic Plants during the 6th—2nd Centuries Bc in Sardinia, Italy. Veg. Hist. Archaeobotany.

[74] Sefc K.M., Regner F., Turetschek E., Glössl J., Steinkellner H. (1999). Identification of microsatellite sequences in *Vitis riparia* and their applicability for genotyping of different *Vitis* species. Genome.

[75] Merdinoglu D., Butterlin G., Bevilacqua L., Chiquet V., Adam-Blondon A.-F., Decroocq S. (2005). Development and Characterization of a Large Set of Microsatellite Markers in Grapevine (*Vitis vinifera* L.) Suitable for Multiplex PCR. Mol. Breed..

[76] Bowers J.E., Dangl G.S., Meredith C.P. (1999). Development and Characterization of Additional Microsatellite DNA Markers for Grape. Am. J. Enol. Vitic..

[77] Thomas M.R., Scott N.S. (1993). Microsatellite Repeats in Grapevine Reveal DNA Polymorphisms When Analysed as Sequence-Tagged Sites (STSs). Theor. Appl. Genet..

[78] Zyprian E., Eibach R., Töpfer R. (2006). Eine neue genetische Karte der Weinrebe aus der Kreuzung “GF. Ga-47-42 × Villard Blanc”. Deutsches Weinbau-Jahrbuch.

