# Supplementary figures and images for: Unearthing Genetic Treasures: Exploring Lost Autochthonous Vitis vinifera Varieties in Lebanon
Source: Genes (Basel). 2024 Dec 17;15(12):1617. doi: 10.3390/genes15121617 (PMC11675416; doi:10.3390/genes15121617)

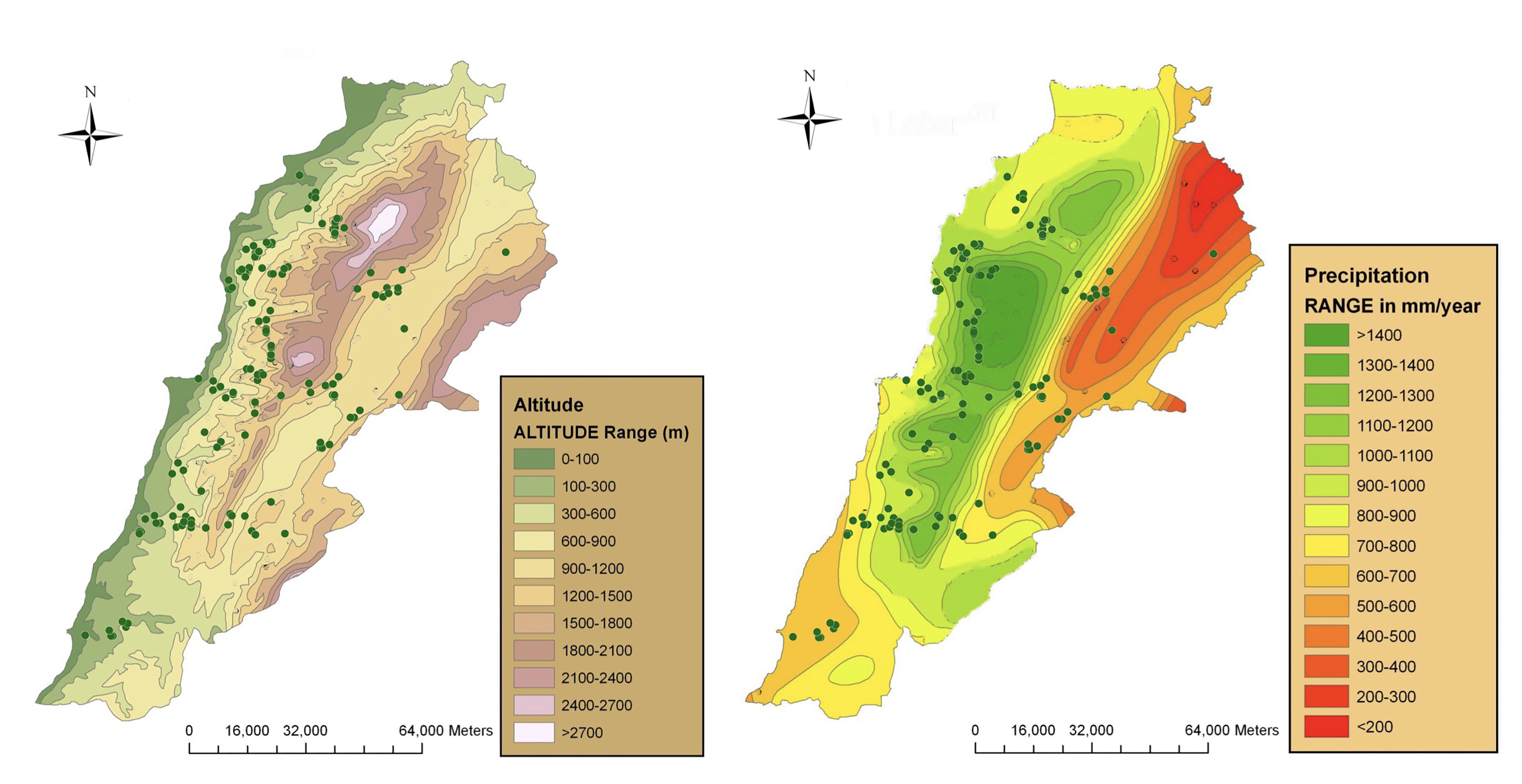

Supplement: Supplementary file 1 [file genes-15-01617-s001.zip › Figure S1. Geographic Distribution of Sampling Sites in Lebanon Based on Altitude in Meters (Left) and Precipitation in mmyear (Right).png]
